# Protein Conformational Dynamics upon Association with the Surfaces of Lipid Membranes and Engineered Nanoparticles: Insights from Electron Paramagnetic Resonance Spectroscopy

**DOI:** 10.3390/molecules25225393

**Published:** 2020-11-18

**Authors:** Elka R. Georgieva

**Affiliations:** Department of Chemistry and Biochemistry, Texas Tech University, Lubbock, TX 79409, USA; elgeorgi@ttu.edu; Tel.: +1-806-834-8166

**Keywords:** protein conformation, protein-surface association, lipid membranes, surface-immobilized protein, EPR spectroscopy

## Abstract

Detailed study of conformational rearrangements and dynamics of proteins is central to our understanding of their physiological functions and the loss of function. This review outlines the applications of the electron paramagnetic resonance (EPR) technique to study the structural aspects of proteins transitioning from a solution environment to the states in which they are associated with the surfaces of biological membranes or engineered nanoobjects. In the former case these structural transitions generally underlie functional protein states. The latter case is mostly relevant to the application of protein immobilization in biotechnological industries, developing methods for protein purification, etc. Therefore, evaluating the stability of the protein functional state is particularly important. EPR spectroscopy in the form of continuous-wave EPR or pulse EPR distance measurements in conjunction with protein spin labeling provides highly versatile and sensitive tools to characterize the changes in protein local dynamics as well as large conformational rearrangements. The technique can be widely utilized in studies of both protein-membrane and engineered nanoobject-protein complexes.

## 1. Introduction

Conformational dynamics is one of the key factors determining protein function [1,2]. Structural transitions, including large interdomain movements and local structure rearrangements, occur upon binding and processing of substrates [3,4], protein–protein interactions [5], and protein–nucleic acid interactions [6], as well as upon binding to the surface of biological membranes [7,8,9,10]. All these structure alterations are relevant to the physiological states of proteins and comprehensive understanding of them is central to developing approaches to monitor and control protein function. On the other hand, under certain not well understood conditions, some proteins adopt misfolded conformation in solutions or upon interaction with lipid membrane surfaces, leading to formation of large aggregates as those found in several neurodegenerative and other diseases [11,12]. Gaining deep understanding of protein conformation and stability is also very important in the cases where these proteins are immobilized on engineered surfaces. Protein immobilization through adsorption or covalent bonding is widely used in biotechnology, protein purification, drug delivery, etc. [13,14]. For such applications, it is important to characterize the efficiency and kinetics of protein immobilization, which significantly depends on the surface properties [15,16]. Furthermore, characterizing the conformational dynamics of proteins upon their immobilization on material surfaces serves to ensure that the protein functional states are preserved; therefore, it is particularly important. Several techniques have been used to infer the structural dynamics of proteins upon association with biologically relevant surfaces, such as exhibited by lipid membranes, but also with engineered surfaces. Such techniques include, for example, FTIR spectroscopy [17], single-molecule fluorescence methods [18], and atomic force microscopy [19].

The emphasis of this review is on the application of electron paramagnetic resonance (EPR) spectroscopy in studies of protein structure and structural dynamics changes upon transition from the solution state to the surface-bound state. EPR in its continuous-wave (CW) and pulse spectroscopy modalities in combination with spin labeling provides the powerful biophysical techniques to study local dynamics and long-range conformational rearrangements of proteins [7,10,20,21,22,23,24,25]. Here, the EPR method with protein spin labeling is briefly described and is followed by the illustrations with relevant applications of this technique to study protein conformational transitions and structural stability upon binding to lipid membranes and engineered surfaces.

## 2. EPR Spectroscopy of Spin-Labeled Proteins

### 2.1. Spin Labeling of Protein Molecules with Nitroxides

**Figure 1:**
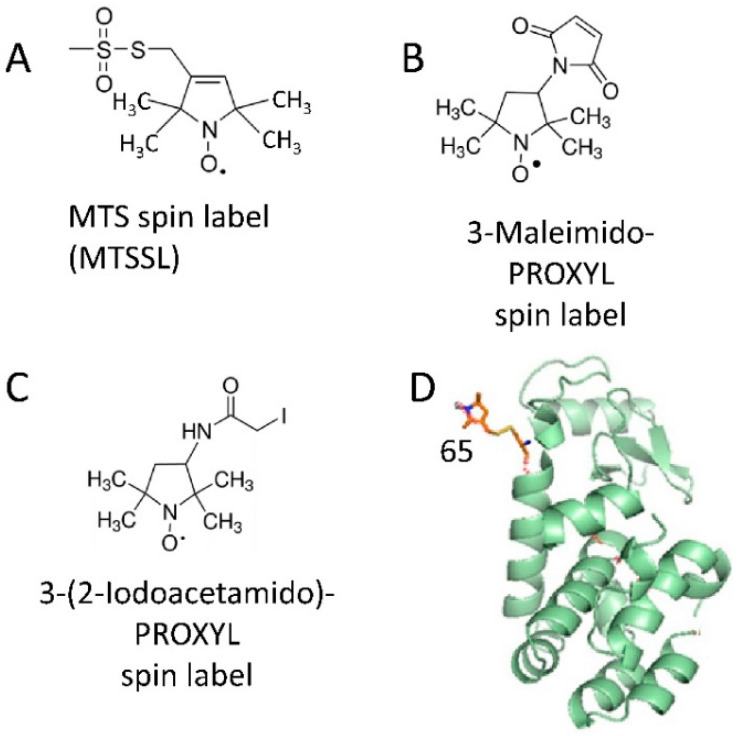
Spin labeling of proteins. (**A**–**C**) show nitroxide spin labels widely used for study of proteins by EPR spectroscopy. (**D**) emphasizes the residue 65 in T4 lysozyme spin-labeled with MTSSL. (PDB accession code 3LZM). Spin-label side chains were generated using the MMM program (Jeschke and Polyhach) [42].

Since EPR spectroscopy detects and studies paramagnetic centers which have unpaired electron spins, its applications to the structure and function of proteins having native paramagnetic centers is restricted to certain classes of metalloproteins [26,27] and radical enzymes [28,29,30]. Thus, protein molecules are predominantly diamagnetic and EPR silent. However, the range of proteins amenable to study by EPR spectroscopy is widely expanded by introducing paramagnetic reporter groups, so-called spin labels, which are covalently attached to desired positions in the protein molecules [20,31,32]. Therefore, EPR spectroscopy can be employed in studies of virtually any protein or protein complex, regardless of its size, environment (e.g., solution, lipid membrane), and flexibility (dynamics), given that it could be spin-labeled. The most universally used spin labels are nitroxides, which in most cases are introduced by covalent attachment of spin labeling reagent to the cysteine residue via disulfide or thioester bond formation. Most often they are mehanothyosulphonate- (MTS), iodoacetamido-, or maleimido-linked nitroxide tags [20,33] (Figure 1A–C). There is a large variety of less often used spin-bearing moieties as well. The cysteine residues used for spin labeling could be endogenous, but overwhelmingly they are introduced at desired positions through site-directed mutagenesis on the background of a cysteine-free protein (Figure 1D). Other types of spin labels based, for example, on trityl radicals [31,34,35], manganese ions [36], copper ions [37,38], gadolinium ions [34,36,39,40] have also been successful. In special cases spin labels are introduced by protein binding to spin-labeled substrate or inhibitor [41].

### 2.2. CW and Pulse EPR Spectroscopy Applied to Spin-Labeled Proteins

Protein-bound nitroxide spin labels have been applied to study protein local dynamics and solvent accessibility at physiological temperatures by means of CW EPR spectroscopy [20,21,24,25,33,43,44,45,46,47]. Additionally, long-range structural rearrangements and local heterogeneities of biomolecules are best determined by using pulse EPR distance measurements between pairs of spin labels in a protein. The experiment is conducted as a rule at cryogenic temperatures but in few cases it was possible at physiological temperatures [7,9,10,20,22,25,28,33,45,46,48,49,50,51].

**Figure 2:**
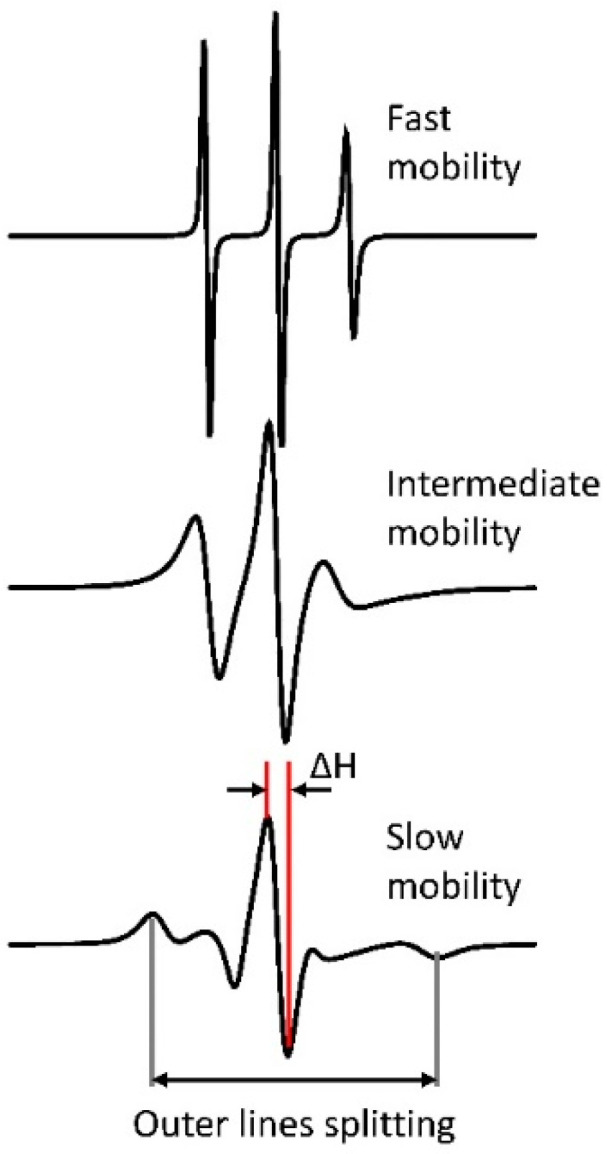
Continuous-wave (CW) electron paramagnetic resonance (EPR) spectra of the nitroxide spin label. The spectra correspond to different ranges of spin-label and protein motion. The coarse parameters, Δ*H*, and splitting of the outer hyperfine lines are shown in the bottom spectrum. Both parameters depend on the extent of spin label immobilization. The increase in the outer line splitting is due to the contribution from the *A*_zz_ hyperfine tensor component. Thus, less mobile spin labels lead to broader spectrum.

In nitroxide-labeled proteins lacking native paramagnetic centers, CW EPR records only the absorption spectrum of the nitroxide radical, usually in the form of the first derivative. The spectrum exhibits characteristic three lines, whose linewidths and amplitudes depend on the intrinsic spin label dynamics caused mainly by the rotational diffusion of the nitroxide moiety in the local potential and to some extent by the protein dynamics in its immediate vicinity as well as the protein global tumbling, segmental motion or chemical exchange [52,53,54]. In general, the frequencies of these motions observed by nitroxide multifrequency CW EPR fall into the range of rotational correlation time (τ_c_) ≅ 10^−12^–10^−8^ s. The anisotropy of hyperfine interaction (hfi) tensor and g-tensor, partly averaged by motions, produces typical asymmetric spectrum with the high-field side being broader. Visually the CW EPR spectrum is broader in the case of spin label with motion that is more restricted (Figure 2). As the diffusion rate decreases from fast to slow motion and is approaching rigid limit, the spectrum changes from three narrow lines with ~15 G splitting, to a more complex shape with broad linewidth until it ultimately approaches the rigid limit spectrum defined by just the magnetic tensors and, when present, by sample anisotropy. In the fast-motion regime, the rate of spin label dynamics is estimated from the central line width (ΔH) and the line intensity ratios. As the spectrum broadens, the outer line splitting in the spectrum (Figure 2) becomes useful for estimating the motional rate. As the motion slows down the spectrum becomes more complex [8,24,45] and may be significantly affected by the local ordering, e.g., microscopic-order-macroscopic-disorder (MOMD) cases [55]. The accurate determination of spin label ordering and dynamics necessitates resorting to sophisticated computational approaches that give rotational diffusion tensor and ordering parameters. This is an advantage to fast motions which yields only the rotational correlation time [55]. Therefore, changes in the spin label environment that produce steric effects, folding and unfolding, or binding to a much larger partner, such as a liposome or surface, all can be assessed through the rich variety of changes in the nitroxide CW EPR spectrum for selected spin-labeled positions. For example, if binding of a partner to a specific protein region decreases the rate of motion of this region, then one will record a CW EPR spectrum having broader lines compared to the EPR spectrum for protein without a binding partner [8,20,45]. Importantly, the motional rates corresponding to the slow-motion regime of spin-labels also depend on the strength of static magnetic field in a way that higher fields lead to more slow-motion spectra [56,57].

The degree of solvent exposure or penetration into the bilayer of certain membrane-bound protein regions can also be assessed by CW EPR spectroscopy. In this case, the residues in this region, with the exclusion of those highly conserved, are sequentially substituted for cysteines and labeled with a nitroxide. Then the CW EPR spectra of multiple spin-labeled cysteine variants are recorded in the presence of a paramagnetic relaxing agent (PRA), such as oxygen, nickel (II)–*N*,*N*1-ethylenediamine-diacetic acid (NiEDDA), or potassium ferricyanide (K_3_Fe(CN)_6_) [45,47,58,59]. The method relies on relaxation enhancement caused by the PRA changing the microwave (MW) power saturation properties of the nitroxide EPR spectrum. The experiment is referenced by one conducted in the absence of PRA. The intensity of the central peak in the nitroxide CW EPR spectrum is recorded as a function of the applied MW power, and the accessibility to the PRA is determined from the obtained power saturation curve [20,45,47,59]. By this method, one can assess the solvent exposure of spin-labeled residue at the membrane surface for certain protein conformations and determine whether this residue becomes more occluded or exposed upon conformational change. Furthermore, for proteins transitioning from soluble to membrane-bound state, one can establish which residues are in contact with the membrane surface or to what depth they may penetrate into the lipid bilayer [8,45,47].

**Figure 3:**
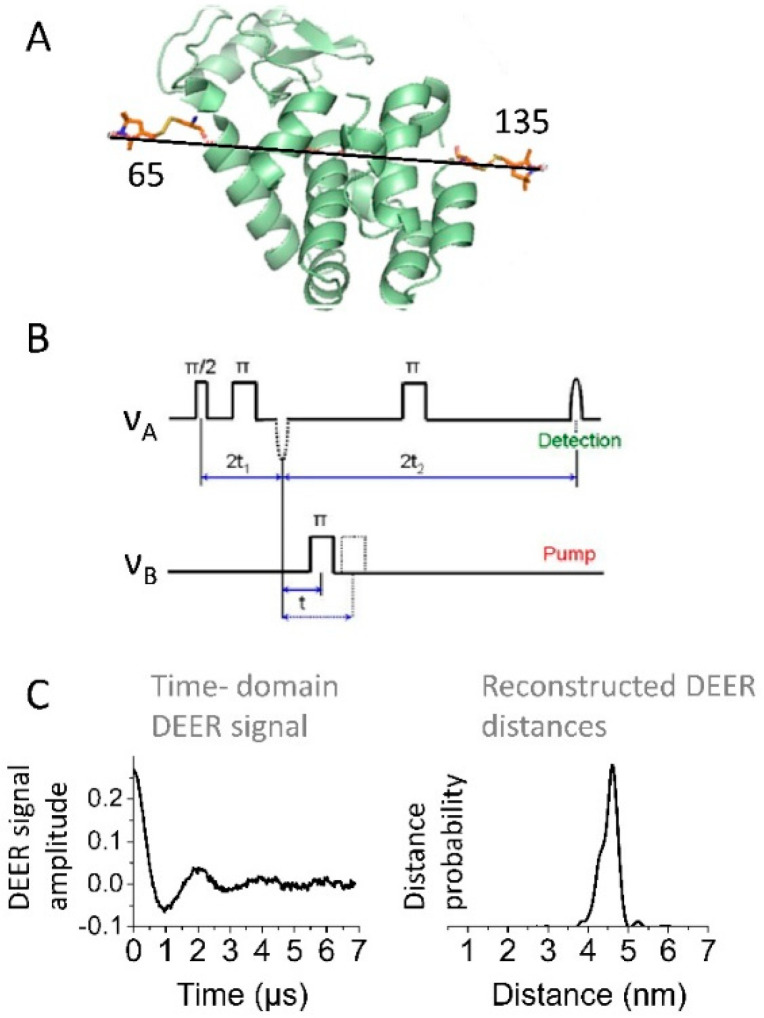
Double electron–electron resonance (DEER) distance measurements in doubly spin-labeled protein molecules. (**A**) The method is illustrated with T4 lysozyme (PDB accession code 3LZM) doubly spin-labeled at positions 65C and 135C. The spin label side-chains were generated with the program MMM. The measured inter-spin distance is indicated by the line. (**B**) The four-pulse microwave (MW) sequence that is used most often in the DEER experiment. The electron spins corresponding to different spin-labeled sites are frequency-selected for detection or pumping, respectively. The three MW pulses separated by fixed intervals select one of the spins to contribute to the detected spin-echo signal. The 4th (pump) pulse applied at a different frequency may select a coupled spin within the respective region of the spectrum. The pump pulse delay is advanced to give the spin-echo amplitude modulation envelope caused by dipolar coupling between the paired electron spins. (**C**) The recorded time-domain DEER signal (left) and the respective distances (right) between the MTSSL spins at residues 65C and 135C are shown. In general, a decaying oscillating time-domain signal is obtained from which the inter-spin distances and distance distributions are reconstructed.

Pulse EPR spectroscopy with spin labeling also encompasses diverse applications in the realm of protein conformational rearrangements. The emphasis here is on the most widely used pulse EPR method, namely the four-pulse double electron–electron resonance (DEER) spectroscopy [22,23,48,60,61,62], which measures distances typically in the range of 2–7 nm and in special cases, e.g., in deuterated proteins, as long as 10 nm or somewhat more [7,63]. Other versions of pulse EPR, such as five-pulse DEER [64] and double quantum coherence [60,65,66] also have been very useful, particularly when increase in sensitivity or distance range was needed. The description of many other recently emerged technological developments in pulse distance EPR spectroscopy is beyond the scope of this review. All these pulse EPR methods measure the strength of the electron spin dipole–dipole interactions between coupled paramagnetic centers (predominantly covalently attached spin labels or intrinsic paramagnetic centers in proteins). The obtained direct information on the coupling strength ν_dd_ of these magnetic interactions (expressed in frequency units) also reports on the distance, *r*, between the paramagnetic spin-labels since ν_dd_ ∝ 1/*r*^3^, (Figure 3). DEER spectroscopy has been used in numerous studies of protein conformation [51,63,67,68,69,70,71,72,73], many of which were carried out on membrane proteins [7,9,10,20,45,46,48,54,74,75,76,77,78,79]. The method is particularly suited for studies of conformational transitions taking place upon binding of protein molecules to lipid membrane surfaces, because very few methods can work well under the conditions of high protein heterogeneity or large flexibility, and the method is unconcerned about the typical large sizes of membrane mimetics. DEER measurements are commonly conducted at cryogenic temperatures. This was shown not to be a matter of concern. Moreover, recent developments of spin labels with longer relaxation times have made it possible to conduct experiments at room temperature [35,80].

## 3. EPR Studies Explore the Conformational Dynamics upon Interactions of Proteins with Biological and Engineered Surfaces

CW and pulse EPR spectroscopy techniques, applied to spin-labeled proteins, are well positioned to detect structural rearrangements that modify fast local and slower long-range dynamics of these complex molecules. Therefore, EPR methods have been extensively used to infer the information on physiologically relevant conformational changes of proteins caused, e.g., by transitioning from their soluble state to the membrane surface-bound state [7,8,9,10,33,45,46,81,82,83,84] but also aggregation and misfolding induced by the membrane surface [82,85,86]. EPR studies have also proved very useful in biotechnology developments for assessing protein stability when immobilized through absorption or covalent attachment to nanosurfaces and synthetic membranes [87,88,89,90,91,92,93]. This range of applications of EPR methods is highlighted with the examples provided below.

### 3.1. Conformational Dynamics Underlying Protein-Lipid Membrane Association

The high capacity of EPR methods to characterize protein dynamics and structural transitions in particularly from the soluble state to the membrane surface-associated state is highlighted here through the studies on human α-Synuclein, tau, and Snf7 proteins.

CW and pulse EPR spectroscopy both have been invaluable tools for studying the conformation changes accompanying the interaction of α-Synuclein with lipid membranes [7,8,10]. α-Synuclein, which is highly abundant in neurons, is a synaptic vesicle-associated protein with its key physiological function associated with the control over the trafficking of these vesicles and the release of neurotransmitters [94,95]. However, under not well-identified and understood conditions, this protein forms large insoluble aggregates found in the patients with severe neurodegenerations [95,96]. Due to the high importance of α-Synuclein in the physiology of the cell and neurodegeneration, a better understanding of the structural aspects underlying its function has been actively sought. However, the methods that can be applied are limited due to the high flexibility of the protein solution form and by the large size of membrane mimetics, further impeded by its structural heterogeneity in the case of the membrane-bound form. Circular dichroism (CD) spectroscopy and nuclear magnetic resonance (NMR) have established that α-Synuclein is unstructured and highly flexible in solutions [97,98]. However, its *N*-terminus and central regions adopt the fold with significant α-helical content upon binding to sodium dodecyl sulfate (SDS) micelles and other membrane mimetics [98,99,100]. EPR spectroscopy was particularly valuable as one of the main methods for overcoming the hurdles encountered by NMR and other biophysical and structural techniques. It provided detailed insights into the conformational rearrangements taking place when this protein binds to the surface of lipid membranes [7,8,10,101]. The studies have been performed on protein-spin label dynamics and on solvent accessibility of multiple singly spin-labeled residues by CW EPR and were advanced by carrying out long-range DEER distance measurements on several doubly spin-labeled α-Synuclein mutants. These CW EPR studies established that on the surface of liposomes, which closely represent the size and morphology of synaptic vesicles, the positively charged *N*-terminal and uncharged central region (also called the non-amyloid component, NAC) of α-Synuclein form an extended uninterrupted amphipathic α-helix. One side faces the solution environment, and the other side is associated with the liposome polar region [7,8,10,102]. This property was also determined for the α-Synuclein mutants found in patients with the familial form of Parkinson’s disease [7]. EPR results have suggested that electrostatic and hydrophobic interactions stabilize the α-Synuclein-membrane complex [8,102]. The capacity of DEER spectroscopy to measure long distances up to nearly 9 nm in membrane-associated proteins, as well as the highly deuterated protein and lipids used in these studies made it possible to observe and characterize the extended helix nature of the binding *N*-terminal part of this protein [7,10]. In another DEER study on SDS and 1-palmitoyl−2-hydroxy-sn-glycero−3-phospho-(1′-rac-glycerol) (LPPG) micelle-bound α-Synuclein, it was found that the protein adopts the U-shape structure formed of two antiparallel amphipathic α-helices connected via an ordered linker [74], thereby confirming the NMR structure of the SDS-bound protein [100]. Next, EPR studies have revealed that the conformation of membrane-bound α-Synuclein can interconvert between the U-shaped antiparallel helices and the single extended helix. The structure depends on the protein-to-lipid (detergent) ratio. The high protein abundance promotes the U-shaped conformation and vice versa [7,75,103]. Thus EPR spectroscopy investigation on multiple spin-labeled single and double cysteine mutants of α-Synuclein, carried out on a variety of lipid membrane conditions, has culminated in a comprehensive characterization of the dynamic nature of this protein and highlighted its versatility conferred by adopting multiple membrane-bound conformations. This structural flexibility is likely much needed for the diverse physiological functions of α-Synuclein, but perhaps under yet unspecified conditions, the membrane association could trigger α-Synuclein misfolding and, possibly, aggregation [104]. It was further found by EPR that the *N*-terminus of membrane-associated α-Synuclein coordinates one equivalent of Cu^2+^ without any observed effect on protein helical structure, suggesting that Cu^2+^ uptake may be relevant to or be a part of α-Synuclein function [105]. Since Cu^2+^ is paramagnetic, the ion coordination was monitored by recording its CW EPR spectrum. The protein site of Cu^2+^ coordination was identified as Met1-Asp2 by using pulse EPR, namely electron spin echo envelope modulation, ESEEM, which reports on unpaired electron hyperfine interactions with the neighboring nuclei [106].

**Figure 4:**
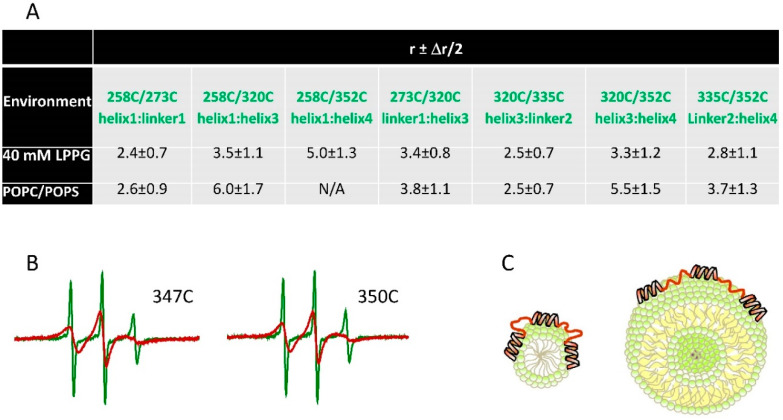
The structure of membrane-bound tau protein as revealed by EPR spectroscopy is shown [45]. (**A**) Long inter-spin distances were measured by DEER spectroscopy for spin-labeled residues located in microtubule binding domain (MBD) repeats. The spin-labeled residues and their locations are indicated in the second row of the table. The lipid membrane mimetic (environment) in which the distances were obtained is indicated in the first column. The obtained distances (*r*) suggest that tau MBD has distinct structures when bound to LPPG micelles and 1-palmitoyl−2-oleoyl-glycero−3-phosphocholine/1-palmitoyl−2-oleoyl-sn-glycero−3-phospho-L-serine (POPC/POPS) liposomes. Longer distances in liposomes suggest more extended conformation. The distance distributions with relatively broad full widths at half maximum Δ*r* indicated highly heterogeneous conformation of membrane-bound tau. (**B**) The binding of tau to lipid membranes was confirmed by CW EPR measurements. Nitroxide EPR lineshape broadening was observed for liposome-bound proteins (red spectra) compared to protein in solution (green spectra). The spin-labeled residues are indicated. (**C**) Model of tau-lipid membrane interaction based on the results from CW and DEER experiments was proposed—tau MBD structure when bound to micelles (left) and liposomes (right) is shown.

Conformational transitions upon binding to lipid membrane surfaces for another highly dynamic and physiologically important human protein tau were characterized using CW and pulse EPR spectroscopy [45]. A key physiological role of tau is to bind and stabilize microtubules, with the tau-microtubule association-dissociation equilibrium being regulated by tau phosphorylation [107,108]. However, when tau is hyperphosphorylated it transitions irreversibly into β-sheet structures that form large insoluble aggregates implicated in severe neurodegenerations [20,109]. In addition to its role in microtubule assembly and stabilization, this protein interacts with cellular membranes [110] with the physiological implications that are currently very little understood. The interaction with membranes in vitro was also linked to tau misfolding and aggregation [111], being in agreement with the detecting membrane-associated tau filaments in vivo [112]. Strikingly, tau associates with microtubules and lipid membranes using the same amino acid region known as microtubule binding domain (MBD). This domain consists of three or four imperfect repeats, depending on the protein isoform [113]. NMR and CD spectroscopies determined that this protein region, similarly to α-Synuclein, is highly unstructured in solution [114], but acquires helical conformation in the presence of membrane mimetics [115,116]. Yet, no clear understanding existed as to how the protein restructures upon binding to the membrane, as well as what its location is with respect to the membrane surface. These questions were answered in the EPR spectroscopy study [45]. Using CW EPR, multiple single spin-labeled mutants with cysteine residues spanning the entire tau MBD were scanned to determine which segments bind to liposomes and adopt a helical structure. To this end, EPR has determined the exposure of these spin-labeled cysteine residues to PRA in solvent/lipid accessibility measurements, as well as the alterations in the CW EPR spectrum lineshape underlying the spin label/cysteine residue dynamics. It was recognized that indeed tau associates with the membrane surface through its MBD and upon lipid binding this region transitions from highly dynamic to significantly more ordered conformation in which short α-helices are formed within each of the MBD repeats. Doubly spin-labeled mutants in the tau MBD were subjected to DEER spectroscopy to measure long-range distances between MBD repeats. The results, under the experimental conditions of this study, strongly suggest that the membrane-associated α-helices in the tau MBD are well separated, thus forming an extended structure, and these helices are connected via flexible linkers. Because no aggregation was observed, it is believed that the observed structures are physiologically relevant. The powerful combination of CW and pulse EPR methods provided a detailed characterization of the lipid-bound tau structure (Figure 4) [45], which provided the first comprehensive insights into how the large conformational dynamics in the tau MBD play a significant role in accommodating its interactions with its binding partners. Indeed, this is currently the most detailed structure of the tau MBD when bound to a physiological partner, and is deemed to be relevant to the structure of the tau protein in the microtubule-associated state.

In addition to intrinsically disordered proteins, which transition into significantly more defined partially folded conformations when peripherally bound to the membrane, EPR methods have been applied to elucidate the structural dynamics of more structured soluble proteins whose conformations drastically change to enable their association with membrane surfaces and possibly facilitating further insertion into the membrane [9,117,118]. Emphasized here is the study by DEER spectroscopy distance measurements and other biochemical and structural biology methods on the Snf7 protein from ESCRT-III (the endosomal sorting complexes required for transport) protein complex [9]. ESCRT III is a conformationally dynamic hetero-oligomer comprised of multiple units on the membrane. The complex plays essential role in membrane remodeling to support vital cellular processes [119,120,121]. Interestingly, each of the hetero-monomers is inactive in solution and possibly undergoes structural adjustments that enable protein-protein and protein-membrane surface interactions leading to formation of large spiral filaments. This conformational activation was spectacularly captured and characterized by DEER spectroscopy for the Snf7 protein [9]. In this study, DEER distance measurements were conducted on multiple single and doubly spin-labeled cysteine mutants of the Snf7 monomer. Experiments were performed for protein in solutions as well as in the presence of liposomes. The measured inter-spin distances within the Snf7 monomer provided direct evidence for protein restructuring. It was established that a certain region (helix 3) is highly dynamic in solutions, sampling between a conformation in close proximity to the core protein domain as well as more open conformation further away from the core domain. However, upon association with the liposome surface, helix 3 exclusively adopts the open conformation. This particular conclusion was based on the measured DEER distances and distance distributions for the pair of spin labels located in the helix 3 and in the core region of Snf7. Broad distance distributions were found in solutions, but they transformed to longer narrowly distributed distances for the lipid-bound protein. It was further found, based on the observed periodic DEER distances between single spin labels in the Snf7 monomer, that on the surface of the liposome, this protein forms filaments with ca. 30 Å periodicity. Thus, the study provided important insights into the structure dynamics underlying the mechanism of the activation of the Snf7 protein.

### 3.2. Conformational Dynamics and Structural Stability Underlying the Immobilization of Proteins on Engineered Surfaces

As pointed out above, the immobilization of functional proteins is of keen interest to technological developments in the biotechnological industries. To this end, proteins are immobilized through adsorption or covalent binding on the surfaces of engineered nanomaterial particles and synthetic membranes [58,89,90,91,92,93,122]. However, in some cases, there could be no compatibility between the surfaces (e.g., between the protein and pore sizes or due to charges or hydrophobicity), which can result in destabilization of the protein functional state leading to partial or the complete loss of function. The environmental factors, such as pH, could also affect protein binding and stability [123,124,125] Therefore, the need exists for robust methods of assessing the protein conformational changes associated with the immobilization on the surfaces of such materials. Provided here are the examples of how EPR spectroscopy helped in such assessments.

Berliner et al. applied the EPR method to study the conformations of trypsin active-site for protein in solution and when it is bound to porous glass [89]. In so doing, a spin-labeled compound 1-oxyl−2,2,6,6-tetramethyl−4-piperidinyl methylphosphonofluoridate, which act as trypsin inhibitor, was used. The mobility of this spin-labeled inhibitor when bound to the protein was studied using CW EPR spectroscopy. The CW EPR spectra recorded for protein in solution indicated the intermediate motional range of the spin label. A small additional spectral broadening was observed for the spin label bound to the immobilized enzyme due to the slower tumbling rate of the protein-glass bead complex. Importantly, identical EPR spectra were obtained for the immobilized protein interacted with the spin-labeled compound either before or after the immobilization. This suggests that the active site conformation in the immobilized trypsin is well preserved [89]. Certainly, this study spearheaded the applications of EPR spectroscopy to testing the structure of immobilized enzymes. Similar conclusions of having unaffected the active site of glutamate dehydrogenase immobilized on sepharose (a crosslinked, beaded-form of agarose) support were also drawn based on the results of the EPR study [93].

**Figure 5:**
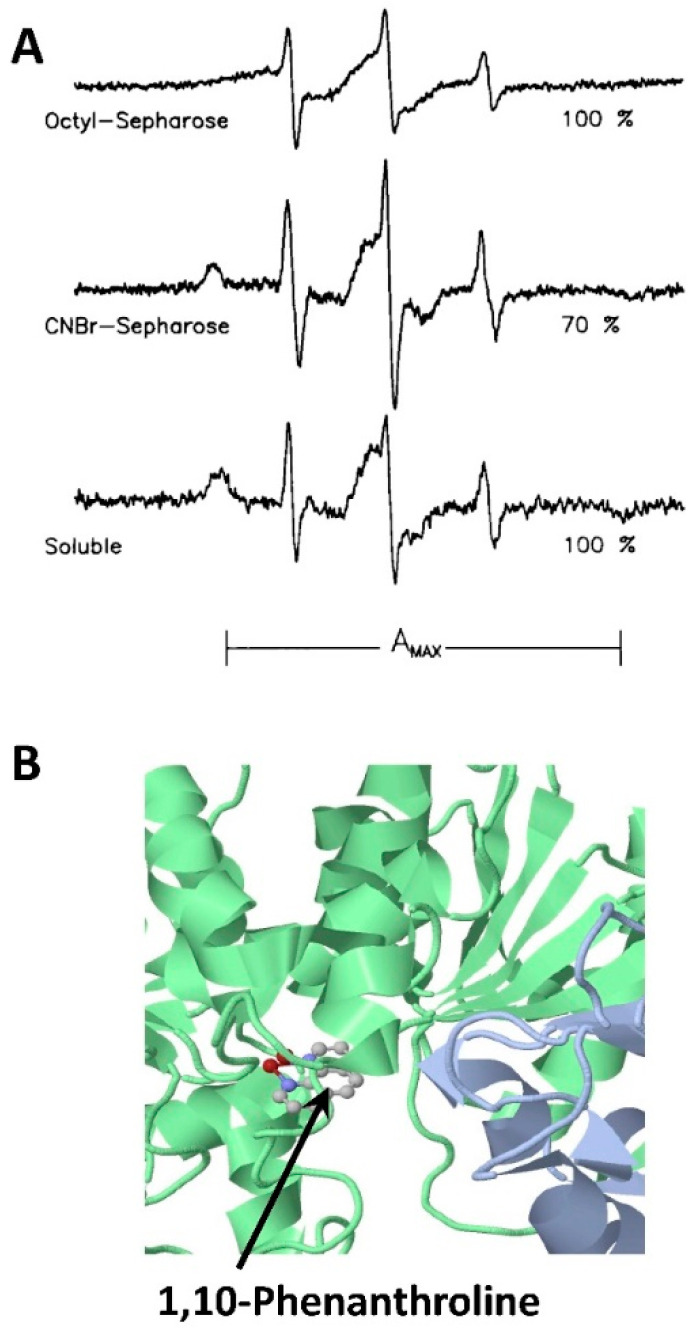
Conformations of liver alcohol dehydrogenase (LADH). (**A**) CW EPR spectra of spin-labeled OPSL bound to LADH in solution (bottom) and immobilized on two different carriers (mid and top). Tree sharp lines in each spectrum are from OPSL in solution with the rest of each spectrum is due to enzyme-bound OPSL. The numbers on the right represent the percentages of catalytic zinc ions and consequently of active sites available to OPSL. A_MAX_ corresponds to the outer spectral splitting, as indicated. (This figure from Ref. [126] is reproduced with permission from John Wiley; Sons) (**B**) A close-up view of 1,10-Phenanthroline bound to LADH at a putative binding site for OPSL, (PDB accession code 5VJ5).

Clark and colleagues applied extensively CW EPR spectroscopy to study the effects of immobilization on the conformational dynamics of enzymes and enzyme active sites [90,91,92,126]. The technique was applied to horse liver alcohol dehydrogenase (LADH) to find the relationship between protein activity and its structure [126]. LADH, an oxoreductase homodimer, catalyzes the interconversions between aldehydes and alcohols and is active toward a broad spectrum of alcohols, aldehydes, and ketones. Therefore, the efforts have been made to understand how to optimize its application to industrial biotechnology. The effect of immobilization was studied [126] for two surfaces, namely CNBr-Sepharose (via covalent attachment) and Octyl-Sepharose (by adsorption). It was found that the enzyme-specific activity has decreased significantly upon immobilization, as compared with the enzyme in a solution; and the decrease was greater in the case of Octyl-Sepharose. Furthermore, the Octyl-Sepahrose-immobilized LADH had reduced thermal stability. CW EPR measurements on the spin-labeled enzyme helped to explain these results. In this regard, two approaches were utilized. Native zinc-coordinating cysteine residue at the catalytic site was labeled with an iodoacetamide-based nitroxide spin label, and the CW EPR spectra were recorded for the protein in free state in solution and when immobilized. Strikingly, two-component CW EPR spectra were recorded for both LADH in solution and immobilized LADH. Spectral decomposition was applied, which determined that the fractions of components corresponding to the slower and faster spin label motion were ca. 75% and 25%, respectively. Based on this, it was discovered that although LADH is a homodimer, with each protomer having one tightly coordinated zinc atom, the protomers in some LADH dimers adopt distinct conformations. Furthermore, the immobilization did not affect the populations of these states for both surfaces [126]. However, after the CW EPR spectra were decomposed, it become apparent that the spectral components for soluble and immobilized enzymes were not identical, with greater spectral broadening observed in the case of immobilized proteins. Thus, it was established that immobilization indeed affects the structure of LADH, but these structural changes were independent of the immobilization method. Based on the EPR data, the reduced thermal stability of the immobilized enzyme was interpreted as a result of conformational destabilization at the active site. For distinguishing between the two immobilization surfaces, a paramagnetic analog of 1,10-phenantroline (OPSL), which coordinates directly to the zinc atom at the LADH active site, was further utilized. It was found that the CW EPR spectrum of OPSL bound to the enzyme in solution was very close to those in the CNBr-Sepharose-immobilized enzyme (Figure 5). However, only 70% of OPSL was bound to the immobilized enzyme, which indicates that 30% of the active sites were inaccessible. Strikingly, a substantially different spectrum was recorded in the case of the Octyl-Sepharose-immobilized protein, providing evidence for an altered active site. These structural insights helped with the understanding of the structural reason underlying the reduced enzyme activity of immobilized LADH.

CW EPR spectroscopy was also used to study the conformation of the papain enzyme, a cysteine protease, which was covalently immobilized on a fully hydrated porous polysulfone membrane [127] Native cysteine residue located in the cleft of the enzyme active site was labeled with an MTS-based nitroxide spin label and the CW EPR spectra were recorded on the spin-labeled protein in solution and when bound to the membrane. For the immobilized enzyme, these spectra consisted of two components with outer hyperfine splitting (which measures the spectral broadening and reports on the spin label/protein dynamics, Figure 2) of 60 G and 70 G, corresponding to an active site with faster and slower dynamics. The hyperfine splitting of 60 G was close to that of papain in solution (53 G). Nevertheless, both conformations in immobilized papain were different from those of free enzyme in solution, an indication that the immobilization indeed alters the structure of this protein. It was further found that the population of the less mobile component increased upon protein denaturation under varying pH, urea, or temperature conditions, as the larger hyperfine splitting of the 70 G component dominated the CW EPR spectrum. Thus, it was concluded that the second broader spectral component originated from protein, which was denatured due to binding to and immobilization onto the membrane. Next, for immobilized papain, experiments conducted to determine spin label accessibility to PRA K_3_Fe(CN)_6_ resulted in the paramagnetic broadening of only the more mobile component of the CW EPR spectrum. This indicates that this component corresponds to the functional enzyme, where the active site is solvent accessible. Therefore, the results from the CW EPR study were instrumental for understanding how and the extent to which the functional conformation is preserved when the papain enzyme is immobilized on an engineered membrane.

In more recent studies, pulse EPR distance measurements were conducted on sepharose-immobilized T4 lysozyme (T4L) with the goal of developing the applications of the method to studies at near-physiological temperatures [35]. To this end, double cysteine mutants of T4 lysozyme were labeled with a triarylmethyl (TAM)-based spin label. The spin label selection and protein immobilization provided the needed increase of the phase relaxation time and prevented from the rotational averaging of the anisotropic dipole-dipole interaction. This beneficial selection of experimental conditions made it possible to measure inter-spin distances in solutions at room temperature. This method could be further extended to study the long-range conformational stability of immobilized proteins relevant to technological developments in the enzyme industry, protein purification, etc.

## 4. Conclusions

EPR spectroscopy of spin-labeled proteins has demonstrated its effectiveness in characterizing the conformational states of proteins bound to physiologically relevant surfaces, such as those of lipid membranes. It also encompassed studies on protein binding to engineered surfaces, such as porous nanocarriers or synthetic membranes. In both cases, valuable information about functional protein conformations can be obtained. However, in contrast to rapidly expanding applications of this method in membrane protein structural and functional biology, its use in characterizing proteins for biotechnological or other industry and laboratory applications is lagging behind. These applications should be further developed to fully benefit from the high sensitivity of the CW EPR to changes in the local protein environment. This is possible through the measurements of local dynamics and solvent accessibility at the protein sites of interest. Furthermore, pulse EPR distance measurements offer an unparalleled capacity to provide deep insight into global protein stability and functionally relevant structural alterations.
